# Noncoding-RNA-Mediated Regulation in Response to Macronutrient Stress in Plants

**DOI:** 10.3390/ijms222011205

**Published:** 2021-10-18

**Authors:** Ziwei Li, Peng Tian, Tengbo Huang, Jianzi Huang

**Affiliations:** 1 Guangdong Provincial Key Laboratory for Plant Epigenetics, College of Life Sciences and Oceanography, Shenzhen University, Shenzhen 518055, China; liziwei1989@126.com (Z.L.); tianpeng151104@163.com (P.T.); tengbohuang@szu.edu.cn (T.H.); 2 Key Laboratory of Optoelectronic Devices and Systems of Ministry of Education and Guangdong, College of Physics and Optoelectronic Engineering, Shenzhen University, Shenzhen 518055, China

**Keywords:** microRNA, long noncoding RNA, circular RNA, macronutrient, omics data

## Abstract

Macronutrient elements including nitrogen (N), phosphorus (P), potassium (K), calcium (Ca), magnesium (Mg), and sulfur (S) are required in relatively large and steady amounts for plant growth and development. Deficient or excessive supply of macronutrients from external environments may trigger a series of plant responses at phenotypic and molecular levels during the entire life cycle. Among the intertwined molecular networks underlying plant responses to macronutrient stress, noncoding RNAs (ncRNAs), mainly microRNAs (miRNAs) and long ncRNAs (lncRNAs), may serve as pivotal regulators for the coordination between nutrient supply and plant demand, while the responsive ncRNA-target module and the interactive mechanism vary among elements and species. Towards a comprehensive identification and functional characterization of nutrient-responsive ncRNAs and their downstream molecules, high-throughput sequencing has produced massive omics data for comparative expression profiling as a first step. In this review, we highlight the recent findings of ncRNA-mediated regulation in response to macronutrient stress, with special emphasis on the large-scale sequencing efforts for screening out candidate nutrient-responsive ncRNAs in plants, and discuss potential improvements in theoretical study to provide better guidance for crop breeding practices.

## 1. Introduction

Nutrient stress is one of the environmental adversities commonly encountered by plants. A thorough understanding of the adaptive strategy to various nutrient stresses will substantially strengthen the theoretical basis for plant breeding practices. Among 14 essential mineral nutrient elements for plant growth and development, six macronutrients, including nitrogen (N), phosphorus (P), potassium (K), calcium (Ca), magnesium (Mg), and sulfur (S), are required in relatively large amounts [1]. Although all these macronutrient elements are mainly absorbed from soil, they differ in sensing and transport pathways, as well as structural and metabolic functions, and may therefore induce different plant responses upon excessive or deficient nutrient supply.

N is the major component of amino acids, nucleotides, chlorophyll, vitamins, and alkaloids, with inorganic ammonium and nitrate being its main form for plant uptake [2]. Both insufficient and excessive levels of N can affect diverse aspects of the plant life cycle. For example, N deficiency could disturb the synthesis of chlorophyll and then reduce the level of photosynthesis, leading to decreased crop yield and quality [3], while an excessive application of N fertilizer might result in a decrease of sugar content [4]. Moderate N deficiency stimulates lateral root growth [5], and severe N deficiency inhibits lateral root growth [6]. P is another essential element involved in vital processes such as photosynthesis, respiration, signal transduction, and nucleic acid synthesis, and it can only be obtained from soil in the form of inorganic phosphorus (Pi) [7,8]. Pi deficiency could not only induce phenotypic changes including dark purple leaf and stem, reduced shoot, and more complex root growth [9], but also cause metabolite alterations such as reduced content of soluble sugar and increased content of organic acids and pigments [10]. K is the third most important macronutrient, and is primarily absorbed by plants in the form of K^+^. It mainly acts on plants via maintenance of cellular osmotic pressure, adjustment of enzyme activity, optimization of photosynthesis performance, and promotion of assimilation product transport [11]. The impacts of K deficiency on plant yield and quality have also been well documented in a variety of species [12,13,14,15,16,17,18]. Ca is a constituent of cell walls, and mainly participates in maintaining the cell physiological state in plants [19]. It is absorbed by plants in the form of Ca^2+^, a well-known second messenger in cellular signal transduction. A lack of Ca during the fruit ripening process might lead to leakage of cell membranes, irregular softening of cell walls, and abnormal fruit development [20]. Mg is involved in enzyme activation, cell homeostasis, membrane structure stability, active oxygen metabolism, nucleic acid metabolism, and signal transduction [21,22,23]. When Mg deficiency occurs in soil, plant growth is restricted, the leaf becomes yellow, and the biomass allocation between organs changes [24,25,26]. On the other hand, an excess of Mg could result in enhanced and weakened carbon metabolism in roots and leaves, respectively [27]. S is a component of amino acids, sulfated polysaccharides, sulfolipids, and vitamins [28], and plays a decisive role in the structure and biological activity of coenzymes and secondary metabolic products [29]. Plants mainly absorb S from soil in the form of inorganic sulfate through sulfate transporters. S deficiency can also lead to metabolite changes such as increased content of total phenol and reduced content of carotenoid in fruits [30,31].

Among the complex regulatory network underlying plant response to environmental stimuli, noncoding RNAs (ncRNAs) are a class of molecules that play critical roles in coordinating nutrient supply and plant demand. Currently, the most widely studied ncRNAs are microRNAs (miRNAs), which mainly act as negative regulators of their target genes through sequence-specific mRNA cleavage or translational repression [32,33]. In plants, miRNAs have long been known to participate in a wide range of biological processes indispensable for plant growth and stress responses [34,35]. Another subclass of ncRNAs that have been recently recognized as regulators for plant responses to biotic and abiotic stresses are long ncRNAs (lncRNAs) [36], which can either be processed into miRNAs to further modulate the expression of downstream genes, or function as molecular decoys to sequester small RNAs from their target RNAs [37]. In addition, there have also been sporadic reports on the potential involvements of other types of ncRNAs such as circular RNA (circRNA) and cis-natural antisense transcripts (cis-NATs) in nutrient stress response [38,39].

The rapidly developing high-throughput sequencing technology has given birth to the explosively growing ncRNA data, which has in turn facilitated the identification and functional characterization of nutrient-responsive ncRNAs in plants. In this review, we will provide an overview of current findings on the ncRNA-mediated regulation in response to macronutrient stress, with a special focus on miRNAs. We will also summarize the large-scale sequencing efforts for screening plant nutrient-responsive ncRNAs, and will discuss possible aspects to be further improved in generating and analyzing ncRNA-Seq data for practical application.

## 2. miRNA-Mediated Regulation in Response to Macronutrient Stress

Under nutrient stress, miRNA may be either upregulated or downregulated, and hence strengthen or relax its inhibition of target gene expression to adapt to variations in environmental nutrient concentrations. In most cases, the target genes of plant nutrient-responsive miRNAs may encode the sensor or transporter of a certain nutrient element, or the transcription factor in regulation of nutrient homeostasis. In this section, we will summarize major responsive proteins and genes for each macronutrient, with special interests in those targeted by nutrient-responsive miRNAs.

### 2.1. miRNA Regulation in N Stress Response

There are four protein families, including the nitrate transporter 1 (NRT1)/peptide transporter (PTR) family (NPF), nitrate transporter 2 (NRT2), chloride channel (CLC) family, and slowly activating anion channel (SLAC) involved in N sensing and transport [40,41,42]. The change in environmental N contents is first sensed by NRT1, a transport protein with amphiphilic structures that can switch between phosphorylation and dephosphorylation at Thr101 according to external N concentration [43]. The expression of *MdNPF6.5* could be induced under either low N or high N condition, and overexpression of *MdNPF6.5* increased the adaptability to N deficiency and enhanced N absorption in apple [44,45]. In addition, several transcription factors involved in the regulation of N homeostasis have also been characterized. Among them, the nodule inception-like protein 7 (NLP7), basic leucine zipper 1 (bZIP1), and teosinte branched 1/cycloidea/proliferating cell factor 1-20 (TCP20) directly interact with N-related target genes [46]. N could promote NLP7 to move into nucleus and control various N-starvation-related genes containing PB1 sequence-specific DNA binding sites [46]. In addition, some autophagic proteins are also involved in response to N stress. Overexpressing *MdATG18a* and *MdATG19* could improve the resistance to N starvation in apple [47,48].

**Figure 1:**
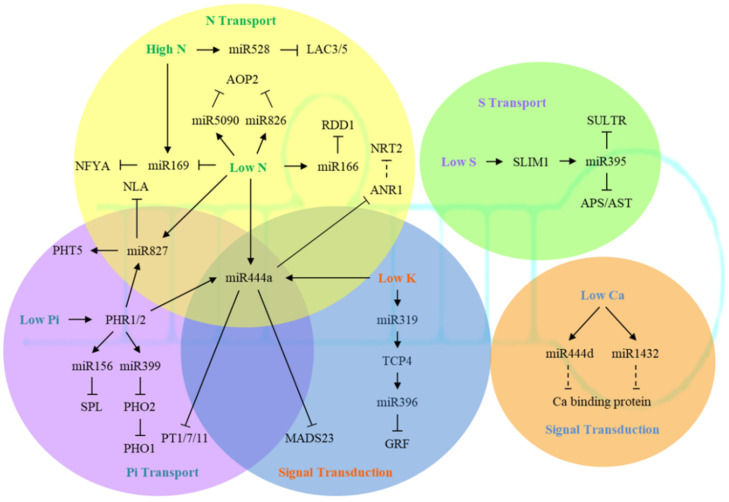
The miRNA-mediated regulatory network for plant responses to macronutrient stress. The miRNAs in the yellow, purple, blue, green, and orange circles are involved in response to stress of nitrogen (N), phosphorus (P), potassium (K), sulfur (S), and calcium (Ca), respectively. Notably, miR827 is responsive to both N and P stresses, while miR444a is responsive to N, P, and K stresses. The dotted line refers to the putative relation between miRNA and its target based on their opposite expression changes. The target genes of nutrient-responsive miRNAs are mainly involved in nutrient transport and signal transduction.

A variety of miRNAs involved in response to N stress have been uncovered and validated in recent studies (Figure 1). Osa-miR166 could target a transcription factor gene, *OsRDD1*, while overexpressing *OsRDD1* induced the expression of genes involved in the uptake process of NH_4_^+^, Na^+^, SO_4_^2−^, Cl^−^, and PO_4_^3−^, thus enhancing N responsiveness and grain productivity of rice [49]. Overexpression of Osa-miR444a, which targeted the MADS-box transcription factor gene *OsANR1*, could increase the accumulation of N and enhance the expression of genes related to N transport under high N environment, but reduce the transport of N from old leaves to young leaves and the adaptability of plants to low N environment [50]. *ZmLACCASE3* (*ZmLAC3*) and *ZmLACCASE5* (*ZmLAC5*) encoding the copper-containing laccases are two targets of monocotyledon-specific Zma-miR528. Transgenic maize plants overexpressing Zma-miR528 had reduced lignin content and rind penetrometer resistance, and were prone to lodging under N-luxury conditions [51]. Intriguingly, two miRNAs, Ath-miR826 and Ath-miR5090, which evolved from an inverse duplication of their common target gene, *alkenyl hydroxalkyl producing 2* (*AOP2*), could both post-transcriptionally suppress AOP2 in Arabidopsis under N starvation [52]. The MIR169 family is also known to be involved in both N and P starvation responses [53,54,55,56,57]. Overexpression of Ath-miR169a inhibited the expression of several nuclear transcription factor Y subunit A (NFYA) genes and accumulated less N in transgenic Arabidopsis [56]. In barley, a potential target gene of Tae-miR169, *TaNFYA-B1*, was found to be induced by N and P to affect root morphology [58]. High N could inhibit the formation of legume rhizobia through the *miR169c-GmNFYA-C-GmENOD40* module, thereby affecting legume uptake and utilization of N in a high N environment [59].

### 2.2. miRNA Regulation in P Stress Response

The phosphate starvation response (PHR) is a type of MYB transcription factor that regulates the expression of phosphate-starvation-induced (PSI) genes by binding to the P1BS motif (GNATATNC) in the promoter region. The expression of *PHR1* is not sensitive to P starvation while regulated by the SPX-domain protein [60]. In a high P environment, AtSPX1 showed a high binding affinity to AtPHR1, which inhibited the binding of AtPHR1 to the P1BS motif of PSI genes; whereas under low P stress, the affinity between AtSPX1 and AtPHR1 was weakened, and AtPHR1 could activate the expression of PSI genes through binding to their P1BS motifs [61]. Among those PSI genes, the members of SPX-MFS subfamily, including phosphate transporter (PHT) proteins, are involved in intracellular P transport process. In case of P deficiency, the expression of *PHT1* was directly induced by PHR1 to promote Pi uptake [62]. Nitrogen limitation adaptation (NLA), another SPX domain protein with E3 ubiquitin ligase activity, can coordinate with the E2 ligase PHO2 to modulate PHT1 degradation, while PHO2 also triggers degradation of PHO1 independent of NLA [63]. PHO1 is an SPX domain protein involved in xylem loading of Pi [64].

The main miRNAs involved in response to P stress are miR399 and miR827 (Figure 1). In Arabidopsis, Ath-miR399 was specifically induced by low P, and it could recognize the *AtPHO2* gene and thereby regulate P homeostasis and signaling pathways in plants [65,66,67]. Currently, miR399 has been found in rice, tomato, alfalfa, kidney bean, and strawberry, showing induced expression by P deficiency stress [66,67,68,69]. At the same time, inhibited expression of PHO2 homologous gene by miR399 was also found in rice and kidney bean under low P conditions [70,71]. Interestingly, long-distance movement of miR399s from shoots to roots was discovered in Arabidopsis, and was suggested to be crucial for enhancing Pi uptake and translocation during the onset of Pi deficiency [72]. Later, miR399 and miR395 were also observed to be phloem-mobile in *Brassica* under nutrient starvation [73]. The function of miR827 was not conserved among different species [74]. Although miR827 was strongly induced under P stress in Arabidopsis and rice, the target gene of Ath-miR827 was *AtNLA*, while the target of Osa-miR827 was *OsPHT5* [75,76]. Similar to miR399, translocation of miR827 and miR2111a between shoots and roots during Pi starvation was also evident in Arabidopsis [77]. In addition, Ath-miR156 and its target, the *AtSPL* (*squamosa promoter binding protein-like*) gene, were also induced by P stress and participated in stress response by regulating the accumulation of organic acids and anthocyanins in the rhizosphere [78].

### 2.3. miRNA Regulation in K Stress Response

Several studies have identified a large number of K^+^-transporting proteins in multiple gene families [79]. Among them, AtHAK5 from the KT/KUP/HAK family may function in both low-affinity and high-affinity transport, and is strongly induced by K deficiency [80,81,82,83]. The expression of *AtHAK5* was regulated by AP2/ERF transcription factor AtRAP2.11, and could also modulate plant response to low K conditions [84]. So far, there have been 71 K^+^ transporters and channel proteins identified in Arabidopsis. These proteins are not only involved in K uptake and transport in tissues and organs, but also are associated with K storage in vacuoles [85,86,87,88,89].

At present, the mechanism of how miRNA directly regulates K uptake in plants is still unclear, but some studies have found that some miRNAs may be involved in K^+^ signal transduction (Figure 1). For instance, while Osa-miR444a was already known to be involved in regulating the accumulation of both N and P, it was also downregulated with its target gene *OsMADS-23* significantly upregulated under K deficiency [90]. In addition, Hvu-miR319 was induced by low K conditions, and then inhibited its target gene *HvTCP4*, which in turn further promoted the expression of Hvu-miR396, thereby repressing the expression of its target gene *HvGRF* in barley [91]. Under low K stress, the miRNAs involved in the regulation of photosynthesis, such as Hvu-miR160a, Hvu-miR396c, and Hvu-miR169h, also displayed differential expression between the two barley genotypes and were suggested to play a key role in low K tolerance [92].

### 2.4. miRNA Regulation in S Stress Response

Transcription factor sulfur limitation 1 (SLIM1) is a global regulator for plant response to S insufficiency. AtSLIM1 can identify the promoters of S-induced genes through a 20 nt consensus UPE-box sequence for transcriptional regulation [93]. In S insufficient situations, plants could activate the high-affinity sulfate transporter genes, *AtSULTR1;1* and *AtSULTR1;2*, to uptake S in the rhizosphere [94,95], or induce the expression of *AtSULTR4;1* and *AtSULTR4;2* to release the S stored in vacuoles [96]. The main miRNAs involved in S stress response is miR395 (Figure 1), which targets two genes encoding ATP sulfurylase (APS) and SULTR2;1 for S assimilation and S allocation, respectively [97]. Ath-miR395 might be induced to maintain optimal levels of sulfate transporters and APSs to achieve S homeostasis under low S conditions [98]. Overexpression of Ath-miR395 in Arabidopsis exhibited remarkable downregulation in mRNA levels of its two target genes, and had more accumulation of S in the shoot, but not in the root [98].

### 2.5. miRNA Regulation in Ca Stress Response

Calmodulins (CAMs), Ca^2+^-dependent protein kinases (CDPKs), and calmodulin-like proteins (CMLs) play an important role in sensing and responding to Ca^2+^ signals [99]. Recent studies have uncovered several gene families encoding candidate Ca^2+^-permeable channels, such as cyclic nucleotide-gated channels (CNGCs), glutamate receptor homologues (GLRs), mid1-complementing activity proteins (MCAs), and calcium permeable stress-gated cation channels (CSCs)/reduced hyperosmolarity-induced (Ca^2+^) increase channels (OSCAs) in Arabidopsis [100,101]. In addition to the classical Ca^2+^ pathway, Ca^2+^-binding proteins FaANN5S and FaANN8 were also regulated by Ca^2+^ signals during the ripening process of strawberry [102]. A recent study indicated that a calmodulin-binding protein and an EF-hand binding protein were the targets of Osa-miR1432 and Osa-miR444d, respectively [103]. This result suggested a possible role of miRNA in affecting Ca homeostasis (Figure 1). Such regulation might also occur via miRNAs targeting the transcripts of *CDPKs* [104]. However, further research will be needed to dissect the detailed mechanism for the function of these miRNAs in Ca stress response.

### 2.6. miRNA Regulation in Mg Stress Response

Regulation of Mg homeostasis in plant cells has been advanced by the discovery of Arabidopsis Mg^2+^ transporters (MgTRs). AtMHX, a Mg^2+^/H^+^ exchanger, is the first cloned MgTR located in vascular system that adjusts osmotic potentials, especially in phloem cells [105,106]. Other superfamily-like MGT-type transporters also respond to Mg stress. Among them, AtMGT6 and AtCNGC10 in roots were possibly involved in Mg^2+^ absorption from the rhizosphere under Mg deficiency. Large-scale screening by deep sequencing has identified a number of candidate Mg-deficiency-responsive miRNAs in leaves and roots of Mg-starved *Citrus*, and the possible target genes of these miRNAs have been shown to be involved in plant response to stress and chlorophyll synthesis [107,108]. Although the functions of some candidate miRNAs and their target genes have been verified in Arabidopsis [107], the regulatory mechanisms of these miRNAs in Mg stress response also remain to be further addressed.

## 3. Large-Scale Identification of miRNAs Responsive to Differential Nutrient Availability

Systematic identification of candidate miRNAs involved in plant response to macronutrient stress has been performed in increasing number of species through comparative expression profiling of miRNAs among differentially adapted genotypes or the same genotype under differential nutrient availability, producing large amounts of small RNA sequencing (sRNA-Seq) data (Table 1). Global screening of miRNAs responsive to N starvation was first reported in Arabidopsis, in which miR160, miR780, miR826, miR842, and miR846 exhibited increased expression, while miR169, miR171, miR395, miR397, miR398, miR399, miR408, miR827, and miR857 showed decreased expression upon N stress [57]. Likewise, comparative expression profiling by deep sequencing helped to identified N-responsive miRNAs from shoots and roots of 7-day N-starved rice [50], from leaves and roots of two wheat cultivars subjected to chronic or short-term N stress [109,110], and from shoots and roots of rapeseeds with 0 or 72 h of N-limitation treatment [111].

The early attempts at large-scale identification of potential P-responsive miRNAs were also reported in Arabidopsis (Table 1), in which the expression of miR156, miR399, miR778, miR827, and miR2111 was induced, but the expression of miR169, miR395, and miR398 was repressed upon P deprivation [53,75]. Since then, an increasing number of candidate P-responsive miRNAs have been obtained in other plant species using different strategies. A microarray-based approach successfully uncovered a subset of 57 known plant miRNAs with differential expression in leaves or roots of soybeans grown under P-deficient and P-sufficient conditions [121], while a genomewide mining dependent on sRNA-Seq identified not only conserved, but also novel miRNAs with significantly altered expression in roots or shoots of a P-efficient genotype soybean treated with low P and high P [114]. Recently, 777 differentially expressed miRNAs across different P treatments and soybean genotypes were also screened out by deep sequencing [115]. Not surprisingly, the sequencing-based expression profiling resulted in substantially larger number of candidate miRNAs than the array-based method did for the same species. In addition, systematic screening of P-responsive miRNAs was also achieved in major crops including rice [122], maize [116,117], and wheat [123].

Global identification of K-deficiency-responsive miRNAs was conducted in roots of two barley genotypes differing in low K tolerance, as well as in wheat roots under five periods of low K treatments, generating approximately 9 Gb and 4 Gb of sRNA-Seq data, respectively (Table 1). The former detected 28 miRNAs differentially expressed at both 2 days and 10 days after low K stress for two barley genotypes [91], while the latter found miR9772, miR1120b-3p, miR531, and miR319 displaying differential expression at all time points during the low K treatments, and suggested that these miRNAs were most possibly involved in mediating plant adaptation to K deficiency [118]. Interestingly, high-throughput sequencing was also employed for identifying differentially expressed miRNAs between two transgenic tomato plants, separately overexpressing *SlmiR168a* and *SlAGO1*, to explore downstream miRNAs (miR171, miR384, miR530, miR858, and miR8007) involved in the *SlmiR168*-mediated *SlAGO1A* regulation upon K stress [119].

To investigate Mg-deficiency-responsive miRNAs, two studies from the same research group obtained 146 and 101 miRNAs with induced or repressed expression in leaves and roots of Mg-starved *Citrus*, respectively [107,108]. Similarly, 87 miRNAs differentially expressed during early embryo development of peanut under Ca-deficient and Ca-sufficient conditions were isolated as candidate miRNAs involved in response to Ca stress [120]. As for S stress, there was one study performing systematic screening of candidate S-responsive miRNAs by sequencing a small RNA library constructed from the pooled rapeseed seedlings simultaneously under S deprivation and cadmium stress [124].

High-throughput sequencing has also been utilized to uncover candidate miRNAs involved in crosstalk between multiple nutrient deficiencies (Table 1). A case in point was that deep sequencing of Arabidopsis small RNAs revealed the specific induction of miR169b/c, miR826, and miR395 by carbon, N, and S deficiency, respectively, as well as the common suppression of miR167, miR172, miR397, miR398, miR399, miR408, miR775, miR827, miR841, miR857, and miR2111 by these three nutrient deficiencies [112]. Recently, sRNA-Seq using shoots and roots of sorghum under single N, P, K, and combined NPK deficiencies revealed that the effects of combined NPK starvation were not a simple addition of individual stress [113]. The expression profiles of common and specific differentially expressed miRNAs observed under single and triple deficiencies indicated that P and K deficiencies had little effect on miRNA expression profiles under N deprivation, and the expression of most K-deficiency-responsive miRNAs was also unaffected by N- and P-deficient conditions, whereas the expression of P-deficiency-responsive miRNAs was affected by both N- and K-deficient conditions [113].

Although the basic principle for identifying nutrient-responsive miRNA was to single out candidates with significantly differential expression between or among samples, the calculation method for expression level and the criteria for statistical significance varied among studies. Furthermore, the tissues for sampling also differed among studies, with roots being most frequently used, owing to their high susceptibility to variations in environmental nutrient levels (Table 1). In contrast, there was only one work focusing on the influence of nutrient stress on miRNA abundance in the reproductive tissue of peanut [120]. The lack of progress in achieving nutrient-responsive miRNAs in reproductive tissues might also be attributed to the fact that most of the nutrient-stressed conditions applied in these studies would cause severe symptoms in vegetative tissues and result in failure of flowering or fruit setting. Nevertheless, the results from vegetative tissues still suggested tissue-specific miRNA regulation upon the same type of macronutrient stress. For instance, 13 miRNAs showed similar expression changes in roots and shoots of soybeans under P deficiency, while 6 miRNAs had opposite expression changes in these two tissues [114]. In rapeseed, 11 upregulated and 15 downregulated miRNAs were specifically identified in roots under N starvation, whereas 25 upregulated and 23 downregulated miRNAs were specifically identified in shoots [111].

Another feature of these sequencing efforts for nutrient-responsive miRNA identification was the integrated analyses of multiomics data. For instance, the downregulation of miR169 family members, which were identified as N-starvation-responsive miRNAs in rice based on sRNA-Seq data, could cause the de-repression of *NFYA,* as validated by the strand-specific RNA-Seq data [50]. In the same study, the confirmation of *MADS25* as a novel target gene of osa-miR444a.4-3p, which was also identified as a N-starvation-responsive miRNA by sRNA-Seq, was aided by analyzing the degradome sequencing data of the N-starved rice [50]. Similarly, combined miRNAome and degradome analysis also helped to reveal the involvement of the miR827-NLA pathway in limited N-induced leaf senescence, as well as the involvement of the miR171-SCL6 and miR160-ARF17 pathways in roots grown under N deprivation [111].

## 4. Other Types of ncRNAs Involved in Nutrient Stress Response

In addition to stacks of reports on miRNA-mediated regulation during the processes of plants responding to macronutrient stresses, there are also emerging studies unveiling the regulatory roles of other types of ncRNAs in these processes, either through interaction with miRNAs or by directly controlling the expression of protein-coding genes. An early study in Arabidopsis revealed that lncRNA induced by phosphate starvation 1 (IPS1) contained a motif complementary to the P-starvation-induced Ath-miR399 with a mismatch loop at the expected cleavage site, and thus acting as a sponge soaking up Ath-miR399 to inhibit its cleavage of *AtPHO2* transcript [125]. Recently, another lncRNA, *T5120*, was found to be modulated by both NLP7 and NRT1.1 to regulate N signaling and improve N use efficiency in Arabidopsis [126]. On the other hand, a genomewide survey of candidate N-responsive lncRNAs has been performed through deep sequencing in *Populus* [127], maize [128], and barley [129], while lncRNAs responsive to multiple nutrient stresses have also been explored by deep sequencing in Arabidopsis [130]. Such large-scale sequencing efforts not only demonstrated an interaction network among lncRNAs, miRNAs, and mRNAs, but also helped to pinpoint key lncRNAs responsible for nutrient stress response. For instance, an analysis of RNA-Seq data derived from Arabidopsis roots exposed to low levels of 12 different nutrients revealed *trans-acting siRNA3* (*TAS3*) as a N-responsive lincRNA, which could produce siRNA targeting NRT2 to regulate N transport and root development under low N conditions [131]. A recent study based on high-throughput sequencing of Arabidopsis roots under the treatments of high Ca content or/and a nonpathogenic growth-promoting rhizobacterium also proposed a lncRNA–miRNA–mRNA regulatory network underlying the improved resistance of Arabidopsis to high Ca stress [132].

Another type of ncRNA capable of regulating the function of miRNA and participating in plant response to macronutrient stress is circRNA, which also serves as an efficient miRNA sponge [133]. High-throughput sequencing of circRNAs has been conducted using the leaves harvested from Chinese cabbage at 0, 3, and 6 days after Ca-deficient treatments [38]. Based on the circRNA-Seq data, dozens of circRNAs with significantly differential expression in different Ca deficiency stages were isolated, among which one circRNA was predicted to be a putative sponge for Bra-miR5716 [38]. Likewise, deep sequencing of the roots from two representative soybean genotypes with different P-use efficiency also identified 120 differentially expressed circRNAs across different P levels and genotypes, among which 70 with miRNA-binding sites were suggested as putative miRNA sponges in response to P deficiency [134]. These circRNA-Seq data will greatly contribute to elucidating the circRNA-miRNA-mRNA network for nutrient stress response.

One more case to be pointed out is the involvement of *cis*-NATs, a class of long RNAs either noncoding or encoding proteins, in plant responses to P stress. In rice, *cis-NAT_PHO1;2_* was shown to promote *OsPHO1;2* translation without changing the sequence, expression level, or nuclear export of *OsPHO1;2* mRNA, and thereby affecting P homeostasis and plant fitness [39,125]. However, the detailed regulatory mechanism of *cis*-NATs in response to macronutrient stress still needs further study.

## 5. Conclusions and Perspectives

The roles of ncRNAs, especially miRNAs, in regulating plant responses to nutrient stress have already been studied for all macronutrient elements, but the responsive miRNA-target module and the regulatory mechanism vary among elements and species. Some miRNAs might modulate a crosstalk among multiple nutrient stresses by acting on different targets. Some miRNAs might also interact with other types of ncRNAs such as lncRNAs or circRNAs to counteract macronutrient stress. The genomewide expression data of ncRNAs generated by sRNA-Seq, along with other omics data from the transcriptome, degradome, proteome, and metabolome, have provided comprehensive insights into the ncRNA-mediated networks in response to single or multiple nutrient stresses for several plant species. The candidate nutrient-responsive ncRNAs screened out from these omics data might serve as promising molecules for further characterization of their detailed functions and for future application in crop genetic engineering. For instance, overexpression of miR5090, which was identified as a N-responsive candidate in Arabidopsis by deep sequencing and subsequent comparative analysis, could lead to improved N uptake and enhanced tolerance to N limitation in transgenic plants [52]. Another case in point was that the transgenic tobacco lines overexpressing Tae-miR408, a K-deficiency-responsive candidate in wheat, were also identified from sRNA-Seq data, which showed significantly improved K uptake, biomass, photosynthesis, and reactive oxygen species scavenging relative to the wild-type plants under K deficiency [118].

Although global expression profiling through deep sequencing has shown some tissue-specific patterns for plant nutrient-responsive ncRNAs, most current studies were based on vegetative tissues. Considering that reproductive tissues such as fruits and seeds are the primary sources for human diet and animal feed, more research efforts are needed to unravel the impacts of ncRNA-mediated regulation on reproductive tissues under nutrient stress. As aforementioned, such efforts may be hindered by the retarded growth or infertility owing to the experimental treatments with ultralow macronutrient concentrations that are not comparable to those in field conditions. Meanwhile, seasonal fluctuations of macronutrient contents in soils are also not neglectable [135]. Therefore, special caution is needed when designing nutrient-stressed treatments for studying plant responses in reproductive tissues. In addition, our preliminary work on the effects of P deficiency on tomato fruit quality also revealed significant alteration in miRNA expression during different stages of fruit development. In this sense, sRNA-Seq data from various developmental stages may enlarge the repertoire of nutrient-responsive ncRNAs and present a spatiotemporal, integrated view of the ncRNA-mediated regulatory network for plant responses to nutrient stress. Taken together, a more precise treatment of nutrient deprivation simulating natural environmental dynamics and a more comprehensive sampling strategy taking reproductive tissues and developmental stages into account will be instrumental in bridging the gap between theoretical study and crop breeding practices.

## Figures and Tables

**Table 1:** Summary for large-scale identification of macronutrient-stress-responsive miRNAs via high-throughput sequencing.

Macronutrient Status	Data Quantity	Differentially Expressed miRNAs *	Species/Genotype **	Tissue	Reference
N deficiency	368.1 Mb	9 ↓, 5 ↑	* Arabidopsis thaliana*/Columbia	Seedling	[57]
	93.8 Mb	9 ↓, 13 ↑	* Arabidopsis thaliana*/Columbia	Seedling	[112]
	26.6 Gb	5 ↓, 30 ↑	* Oryza sativa*/Nipponbare	Shoot	[50]
		57 ↓, 15 ↑		Root	
	1.5 Gb	2 ↓, 1 ↑	* Triticum turgidum*/Svevo	Flag Leaf & Spike	[109,110]
		4 ↓, 5 ↑		Leaf & Stem	
		6 ↓, 5 ↑		Root	
	1.5 Gb	2 ↓, 3 ↑	* Triticum turgidum*/Ciccio	Flag Leaf & Spike	
		4 ↓		Leaf & Stem	
		3 ↓, 4 ↑		Root	
	3.4 Gb	71 ↓, 52 ↑	* Brassica napus* /Zhongshuang11	Shoot	[111]
		64 ↓, 37 ↑		Root	
	3.8 Gb	28 ↓, 8 ↑	*Sorghum bicolor*/BTX623	Shoot	[113]
		25 ↓, 13 ↑		Root	
P deficiency	132.6 Mb	21 ↑	* Arabidopsis thaliana*/Columbia	Shoot	[53]
	657.6 Mb	22 ↓, 33 ↑	* Arabidopsis thaliana*/Columbia	Shoot	[75]
		20 ↓, 25 ↑		Root	
	961.9 Mb	27 ↓, 7 ↑	* Glycine max*/BX10	Root	[114]
		40 ↓, 12 ↑		Shoot	
	4.3 Gb	24 ↓, 22 ↑	* Glycine max*/Bogao	Root	[115]
	4.4 Gb	49 ↓, 34 ↑	* Glycine max*/Nannong94-156	Root	
	27.6 Mb	3 ↓, 2 ↑	*Zea mays*/Inbred line 178	Root	[116]
	14.2 Gb	174	*Zea mays*/Inbred line Q319	Leaf & Root	[117]
	4.2 Gb	16 ↓, 33↑	*Sorghum bicolor*/BTX623	Shoot	[113]
		58 ↓, 18↑		Root	
K deficiency	4.4 Gb	22 ↓, 25 ↑	*Hordeum vulgare*/XZ149	Seedling	[91]
	4.5 Gb	21 ↓, 17 ↑	*Hordeum vulgare*/ZD9	Seedling	
	4.2 Gb	7 ↓, 5 ↑	*Triticum aestivum*/Kenong9204	Root	[118]
	3.2 Gb	110 ↓, 122 ↑	*Solanum lycopersicum*/JZ18 vs. *35S:SlmiR168a*	Leaflet	[119]
	3.8 Gb	58 ↓, 102 ↑	*Solanum lycopersicum*/JZ18 vs. 35S:rSlAGO1	Leaflet	
	4.0 Gb	12 ↓, 20 ↑	*Sorghum bicolor*/BTX623	Shoot	[113]
		16 ↓, 6 ↑		Root	
Mg deficiency	2.0 Gb	71 ↓, 75 ↑	*Citrus sinensis*/Xuegan	Leaf	[107]
	1.0 Gb	69 ↓, 101 ↑	*Citrus sinensis*/Xuegan	Root	[108]
Ca deficiency	7.0 Gb	87	*Arachis hypogea*/Baisha1016	Embryo	[120]
S deficiency	101.9 Mb	2 ↓, 2 ↑	* Arabidopsis thaliana*/Columbia	Seedling	[112]

* The symbol ↑ refers to induced expression, while the symbol ↓ refers to suppressed expression of miRNA upon the corresponding macronutrient stress. A number without a ↑ and ↓ symbol indicates the total number of differentially expressed miRNAs. ** Genotype, or ecotype, or cultivar, or line.
